# Gender difference in the effect of cultural distance on academic performance among cross-border students in China

**DOI:** 10.1186/s41155-021-00199-4

**Published:** 2021-10-21

**Authors:** Jieyi Hu, Chau Kiu Cheung

**Affiliations:** 1 School of Humanities, Jinan University, Zhuhai, Guangdong China; 2 Department of Social and Behavioral Sciences, City University of Hong Kong, Hong Kong, China

**Keywords:** Gender, Cultural distance, Academic performance, Cross-border student

## Abstract

Cross-border students’ academic performance draws people’s attention, whereas perceived cultural distance might influence their performance with gender difference. Based on role theory, men and women present different roles in society, and women are good at perceptual, cognitive aspects, making them more sensitive to cultural distance. Finding shows that the negative moderation role of gender existed in the relationship between cultural distance and academic performance. Particularly, female students showed lower cultural adaptation after cross-border migration, which then influenced their academic performance in universities. This study provides implication for policymakers and universities to pay more attention with additional resources to support female students’ cultural adaption.

## Introduction

Migration shapes and changes in receiving societies (International Organization for Migration, 2013), which is crucial in understanding their move, ideals, values, and beliefs with them (Phinney et al., 2001). Migration is one of the most complicated components of demographic change (Bell et al., 2002). Although most of the studies have examined international variation in movements (Guo et al., 2018), few have explored internal migration and constructed a framework related to internal migration or cross-border movement. Internal migration or border crossing may be different from traditional migration. However, internal migration has become more popular than international migration in most countries in recent years (Guo et al., 2018). Mobility is a distinctive feature of cross-border migrants between Hong Kong and Mainland China (Li, 2011). Although Western research has mainly paid attention to international migration (Yang & Qin, 2016; Yue et al., 2016, p.79), this study of cross-border students in universities contributes to filling the research gap about cross-borderers’ wellbeing.

Identities, cultural distance (norms, values, cultures, customs, and differing views), public awareness, ethical sensitivity, and motivation all influence migrants’ life after migration (Sheu & Fukuyama, 2007). When it comes to the issue of migration or cross-border transfer, cultural difference refers to the difficulties faced by immigrants and cross-borderers to integrate into the host society. Bean (1986) agreed with Tinto’s (1993) view that factors beyond the institution exist, such as the environmental factors influencing students’ academic interactions with their teachers and peers, as well as those affecting their social relations in the host society. One of the crucial environmental factors is the cultural factor. Thus, Tinto (1975, 2010) incorporated students’ interaction, performance, and retention in an environmental factor model. Moreover, encouragement and decreasing the cultural distance as cultural factors can strengthen a person’s goal commitments (Nora et al., 1990), improve academic performance (Cabrera et al., 1992), promote persistence, and thus reduce dropout rates (Nora & Cabrera, 1996).

For a cross-border student group, academic performance is likely a distinctive feature influenced by other social experiences after crossing the border (Guo et al., 2018). A student’s academic achievements (Kuncel et al., 2005) serve as a common indicator of academic performance. Cross-border students are likely to integrate into mainstream society by assimilating its language or broader culture (Echenique & Fryer, 2007; Forrest & Kearns, 2001). The student integration model proposed by Tinto (2010) is useful to enhance social and academic connectivity among the students. Here, education is a resource that facilitates integration (Marhuenda, 2017). Beekhoven et al. (2002) related students’ academic performance acculturation in the university or the new environment. While indicating that academic and social integration matter, that theoretical insight does not tell what they would do to achieve academic and/or social integration in their setting (Tinto, 2010). Thus, further research on predictors of academic performance is necessary.

Countless studies have examined the factors of academic performance, especially among cross-border students. Cultural distance might be one of the predictors of academic performance in cross-border student groups. The relationship between academic performance and cultural distance or cultural adaptability has also found support in many studies. For example, Martin et al. (2017) found that the correlation between adaptability and academic buoyancy, which is a form of academic performance, was significantly more positive in the Chinese student than in other. Accordingly, academic performance significantly influences adaptability among Chinese students. Martin et al. (2012, 2013) proposed adaptability to be an important indicator for individuals to successfully deal with the fluctuation changes accounting in an academic area or even economic, cultural, and technological aspects (Hofäcker et al., 2010; Tomasik et al., 2010). Although studies of the relationship between perceived cultural distance and academic performance have become popular (Fiske & Markus, 2012; Johnson et al., 2011; Melkonian et al., 2019), the moderation role of gender remains uncertain.

Cultural distance between social groups has been supposed to be a crucial predictor for intergroup attitudes (Allport, 1954), resulting in intergroup attitudes influencing relationships or ties among people (Wray et al., 2011). Meanwhile, a study reported that multiculturalism can lead to inevitable ghettoization and polarization, which are dangerous cultural phenomena (Penninx et al., 2006). However, male and female students have different cultural adaptations. Female students who are good at perceptual, cognitive aspects are sensitive to new culture and environment, whereas men are likely to interact with others without being shy (Dupuis et al., 2008). Moreover, this data contributes to examine the gender difference in the effect of cultural distance on academic performance among cross-border students. Based on role theory, male and female students perform differently in society. Men have dominated in cultural domains (e.g., science, technology, and athletics; Miller, 1999; Guttmann, 1991, Battersby, 1989). By contrast, women may benefit by displaying talents that are different from those of men (Campbell, 1999; Cashdan, 1996). Therefore, perceived cultural distance might have differential effects on academic performance because of gender. This research studies gender difference in the effect of cultural distance on academic performance among cross-border students in China.

### Effect of cultural distance on academic performance

Educational outcomes typically include three aspects, namely, enrollment, attainment, and achievement (Cuesta et al., 2016; Glewwe et al., 2011; Mitchell et al., 2008; Snilstveit et al., 2017). Considering universities in Mainland China, educational outcomes are more associated with achievement, as enrollment rates are always stable with Hong Kong migrants coming to Mainland China, whereas attainment is greatly susceptible to family education. Therefore, educational achievement is vital, which always presents the Grade Point Average (GPA) (Berthold & Hoover, 2000) and prizes (Kuncel et al., 2005).

The relationship among university students’ grade, cultural adaptability, and academic integration in host society is prominent (Hoffman & Lowitzki, 2005). Students’ social and cultural capital relates to their academic performance in college (Hagedorn & Tierney, 2002; Warburton et al., 2001). Martin et al. (2017) investigated the correlation between academic buoyancy and adaptability (including cultural adaptability) that is significantly higher in Chinese student samples, in which, academic buoyancy is a form of academic performance.

The definition of cultural distance, given by Triandis (1994), concerns difference in the mother tongue, religion, family and marriage life, and values across cultures. Milem and Berger (1997) indicated that students come to an institution with specific original characteristics, where they encounter new experiences with values, ideas, and norms. Then, as they also interact with their teachers and local peers, they develop perceptions and adaptation to the present environment. Cultural distance is also the way they feel when they come home after years of crossing the border. Conflicts may happen to migrant students, resulting in poorer mental health (Covarrubias & Fryberg, 2015), or worst, home–school value conflict, where values in school are contrary to the values in their hometown, leading to lower academic achievement and wellbeing (Vasquez-Salgado et al., 2015).

Drawing from the cultural orientation emergent from four folds of acculturation (Berry, 1997; Bourhis et al., 1997), this study relates cultural distance to academic performance to determine how the distinctive feature among cross-border students influences their academic performance. People encounter cultural distance when they engage with a person or a context with different opinions of appropriate values and behaviors with theirs (Markus & Conner, 2013; Stephens et al., 2007; Stephens et al., 2012). Cultural distance may increase feelings of exception of non-academic integration, resulting in underperformance, dropout, or disengagement of students’ group (Fiske & Markus, 2012; Johnson et al., 2011).

### Gender matters: the effect of cultural distance on academic performance

The predictors of academic performance, especially in migration groups, include age (Gadzella et al., 2002; Jost, 2008; Kotey & Anderson, 2006), ethnicity (Lu et al., 2003), and residency status (Jost, 2008). These predictors of academic performance among migrant or cross-border students are under the effect of cultural distance. These predictors might be the moderators between cultural distance and academic performance. However, although gender also appears to influence migrants’ academic performance (Jost, 2008; Peiperl & Trevelyan, 1997), the mechanism for its influence has remained unclear. The achievement observed for cross-border students between male and female students has prompted this study to fill the gap about the differential effect due to gender.

The effect of cultural distance on academic performance has been presented in the work of Melkonian et al. (2019). However, perceived cultural distance is different between the man and woman. Some studies performed in Western countries have presented that women are more interested and involved in deinstitutionalized spirituality than their counterparts (Heelas & Woodhead, 2005; Stark, 2002; Trzebiatowska & Bruce, 2013), such as cultural adaptation after migration. Gender differences might influence women to adapt to the cultural difference in the host society, such as biological sex differences or personality traits (Francis & Penny, 2014; Thompson, 1991). In addition, same-sex relationships are likely to involve greater reciprocity and emotional intimacy, which are crucial for reducing their perceived cultural distance, than the direct competitions among men (Baumeister & Sommer, 1997; Geary, 2010). Thus, women communicate through indirect means (e.g., gossiping) rather than dispute competition (Campbell, 1999). Moreover, women supposedly use resources for attractiveness and sexual exclusiveness in a new group (Fischer, 2004; Hrdy, 1999). By contrast, on average, cultural displays may be preferable among men because they actively seek avenues for fighting the opportunity to develop it (Kanazawa, 2003). When men present their own culture, they are more challenging with riskier strategies, such as when in sports (Ronay & von Hippel, 2010) and even playing games with a female opponent (Dreber et al., 2010).

Furthermore, some scholars have argued that cultural distance has disadvantages to female students’ academic performance. The cultural displays of men present the function of demonstrating their mental and behavioral talents, which then serve as reliable indicators of culture in the society (de Block & Dewitte, 2009; Lombardo, 2012). Therefore, a male-dominated society can deprive women’s resources (Xie & Shauman, 2003) and self-confidence (Hyde & Kling, 2001), which impedes women’s cultural adaptability and academic performance. By contrast, women have developed some strengths. For example, Hyde (2005) and Spelke (2005) proposed that women are good at perceptual, cognitive aspects, which brings them difficulties in overcoming cultural distance to focus on their courses. Cultural distance and gender intersect in many ways. High cultural distance may involve the consideration of safety, basic survival needs, and welfare (particularly welfare of children), which are mostly concerns of women (Dupuis et al., 2008). In addition, sociocultural factors in gender roles and socialization patterns (Levitt, 1995; Mol, 1985) show that women might be less interactive with their teachers and peers. Female students are more shy than male students in talking and cooperating with others. Furthermore, women are mostly concerned with others in the new environment (Dupuis et al., 2008); thus, their sensitivity may hinder their cultural adaptability.

Given the above discussion, whether female students have higher cultural adaptability is the research question in this study. Female students encounter fewer resources and biased evaluation for cultural adaptability in a male-dominated society. Although the situation has been increasingly improving, the patriarchy history remains. In comparison with the previous literature that uses family (spouse) data, the present work used data on university students to examine the effect of gender in the relationship of cultural distance and academic performance.

### Role theory explaining gender difference between cultural distance and academic performance

Gender roles are lifelong expectations shaped by culture “through direct communication and through media” (Kerr & Multon, 2015). The updated version of role theory has related gender to culture. Cross-cutting social group identities (e.g., gender, race, ethnicity, class, religion, nativity, sexuality) and social contexts (e.g., historical period, country, region) might interact to shape individuals’ gender beliefs and values (Chatillon et al., 2018). Role theory differentiates the roles of men and women in society (Shimanoff, 2009). As such, role theory can predict male and female performance. Men and women behave differently since they supposedly fulfill different roles in society, such as task orientation, dominance, and even independence (Shimanoff, 2009). Role theory has been useful in ample research on communication and interaction (Allen et al., 2002; Dindia & Canary, 2006; Eagly, 1987), which influences cultural distance. Although most men and women communicate in similar ways, researchers have emphasized statistical differences between them (Hyde, 2005; Martell et al., 1996).

**Fig. 1:**
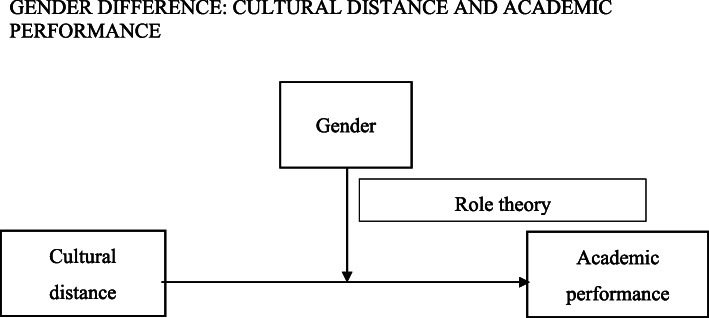
Theoretical framework

Role theory suggests that culture acts on the sorting process rather than on the valuation process, resulting in gender difference in society (Charles & Bradley, 2002; Charles & Bradley, 2009). In this view, women are socialized to choose the fields of study that furnish students with more cultural than economic capital, which makes them feel more sensitive to cultural change (Hakim, 2000). Gender affects returns (e.g., wages). Thus, given that women’s fields provide less economic capital and fewer quantitative skills, they have drawbacks in acquiring resources for cultural adaptation (Paglin & Rufolo, 1990; van de Werfhorst, 2002). Women communicate through indirect means and sexual exclusiveness in a new group, thus hindering the reduction in cultural distance (Fischer, 2004; Hrdy, 1999). Moreover, women are good at perceptual, cognitive aspects in their roles to become sensitive to cultural distance. Because of their sensitivity to and concern for cultural distance, female students cannot focus on their study, resulting in poorer performance than their counterparts (Fig. 1).

Given the above literature review and theoretical framework, we propose the following hypotheses about cross-border students:

- H1: Cultural distance exerts a negative effect on academic performance.
- H2: Cultural distance exerts a negative effect on academic performance, particularly in female students.

## Methods

### Participants

Participants were Hong Kong students studying in universities in Mainland China, in their various grades, classes, and majors. Questionnaires were distributed to these students in Guangzhou at Guangdong Province in Mainland China. As verified by backtranslation (Brislin, 1970), the questionnaire in the present study initially underwent a Chinese translation process. Each potential participant was found by teachers or by convenience sampling. This survey was conducted in the second semester of the academic year. Thus, although first-year students have the academic performance in the last semester, all the collected data were included in this study.

A total of 616 students from Hong Kong studying in Mainland China universities (from undergraduates to graduates) took part in this survey in early 2019. Among them, 40.3% were male, and 59.7% were female. Most of them were in the age of 18–25 (*N* = 587). In addition, the average grade was 2.33. The average time of studying in Mainland China was 99.54 months with a minimum and maximum of 1 and 295 respectively. The entrance exam score was coded 0–4, with an average score of 3.21.

### Measurements

#### Academic performance

GPA (Berthold & Hoover, 2000) and other prizes (i.e., curricular and extracurricular activities) represented academic performance, because they were easily reported as objective indicators. The most obvious subjective approach is the self-reported GPA. However, obtaining the school report of GPA is difficult due to limited research channels. As for academic performance, self-reported GPA and prizes were indicators. GPA and prizes (i.e., the frequency of receiving prizes) both ranged from 0 to 4. They summed to represent a student’s academic performance.

Information on the admission scores and GPA in the recent semester were asked in the questionnaire. Belloc et al. (2010) stated that a student’s academic success in secondary school is directly related to the success of the same student in college. Thus, the entrance exam score should be a control factor in the analysis.

#### Cultural distance

Previous works that have studied students’ interactions in unfamiliar environments (Moschetti & Hudley, 2008; Stanton-Salazar, 1997) presented theoretical explanations that social and cultural capitals accumulate over time (Coleman, 1988). Participants responded to the question, “In the past year, what did you think was the difference between Hong Kong and Mainland China?” on a 10-point scale. As such, the experience was a plausible predictor of academic performance in the recent semester.

#### Grade

Grade ranged from 1 to 8, representing students’ grades from first to higher grades (including the first year, second year of undergraduates or graduates, etc.).

#### Duration of study in Mainland China

The duration of stay in the host society may influence cultural distance between the hometown and host society (Coleman, 1988). It might influence cross-borderers’ perceived cultural distance. Although all the participants were Mainland China university students from Hong Kong, their time of starting their study is a variable. Thus, the duration of study in Mainland China is a control variable.

#### Acquiescence

According to Baumgartner and Steenkamp (2006), “acquiescence” is the variable generated to measure a response set in rating. To control for the probable bias in self-report, the analysis included an acquiescent response set. Bachman and O’Malley (1984) suggested that the proper approach is to average a handful of heterogeneous items in the rating scales. Acquiescence is the average of the average of positive items and the average of negative items to provide weights to the positive and negative sets. It was also the control variable used in the regression analysis in this study.

### Procedure

The consent form included information regarding the purpose of the study, the researcher, and so on. All the participants were informed of the purpose of this study and the process of the survey before the survey started. They then would realize their right to participate or not. Moreover, the participants could stop the survey whenever they feel uncomfortable. All their responses to the questionnaires would remain confidential.

Questionnaires were distributed to Hong Kong cross-border students in Mainland China with diverse universities, grades, classes, and majors by trained researchers. Once the students completed the survey, they would be given gifts.

### Data analysis

Statistical Product and Service Solutions 24.0 was the software used for data analysis. Potential multicollinearity problems were not evident (tolerance < .3). The analysis proceeded with correlation and regression analyses. Regression analysis held academic performance as the outcome, and cultural distance and backgrounds as predictors. Model 2 of the analysis notably tested the moderating effect of gender on the effect of cultural distance on academic performance.

## Results

There were 616 participants in total involved in this study, while after selected those participants were not first-year students. Among them, 40.3% were male (*N* = 248) and 59.7 % were females (*N* = 368). The mean (*SD*) of academic performance, cultural distance, duration studying in the Mainland China, grade, and entrance exam score were 5.13 (1.353), 6.25 (1.941), 99.54 (84.498), 2.33 (1.355), and 3.21 (.323) respectively (Tables 1 and 2).

**Table 1:** Personal characteristics (*N* = 616)

	Coding	*N* (%)	*M* (*SD*)
**Gender**			
Male	0,1	248 (40.3)	
Female	0,1	368 (59.7)	
**Age**			
Under 18	0,1	16 (2.6)	
18–25	0,1	587 (95.3)	
26–34	0,1	7 (1.1)	
Over 35	0,1	6 (1.0)	
**Grade**	1–8		2.33 (1.355)
**Duration studying in the Mainland China**	Months		99.54 (84.498)
**Entrance exam score**	0–4		3.21 (.323)
**Cultural distance**	0–10		6.25 (1.941)
**Academic performance**	0–4		5.13 (1.353)

**Table 2:** Correlations among main variables

	Cultural distance	Academic performance	Duration studying in the Mainland China	Gender	Entrance exam score
Cultural distance	1.000				
Academic performance	−.053	1.000			
Duration studying in the Mainland China	−.131*	.052	1.000		
Gender	−.015	−.005	−.100*	1.000	
Entrance exam score	−.015	−.005	−.100*	.036	1.000

*p < .05. **p < .01. ***p < .001

The correlations between academic performance and cultural distance, between gender and cultural distance, and between gender and academic performance were not significant. Therefore, there is no collinearity problem in the interaction effect of gender and cultural distance on academic performance (Table 3).

**Table 3:** Regression analysis of academic performance

	Standardized coefficients		Tolerance	
Independent variable	Model 1	Model 2	Model 1	Model 2
Cultural distance	−.034	−.024	.932	.927
Gender	.045	.041	.963	.962
Age	−.167**	−.168**	.830	.830
Grade	.332***	.333***	.806	.806
Duration studying in the Mainland China	.019	.015	.933	.933
Entrance exam score	.018	.017	.935	.935
Acquiescence	.116	.100*	.961	.950
Gender × cultural distance		−.141**		.984
*R*^2^	.104	.124		

*p < .05. **p < .01. ***p < .001

The moderating effect of gender on the effect of cultural distance on academic performance was significantly negative (*β* = −.141, *p* < .01), which aligned with the view of Chatillon et al. (2018) that the cross-cutting social group affects values and behaviors, contributing to the research gap of cross-cultural studies. The main effects appeared in model 1, while the interaction effect presented in model 2. Male and female students showed differential cultural adaptation after crossing the border, as supported by van de Werfhorst (2002). Gender as well as cultural distance showed no significant effect on academic performance in both the main effect model (model 1) and interaction effect model (model 2). One of the predictors of academic performance, age, exhibited a significant negative effect on academic performance (*β* = −.167, −.168, *p* < .01). In addition, the student’s admission grades had a significant positive influence on academic performance (*β* = .332, .333, *p* < .01). Acquiescence (*β* = .100, *p* < .05) presented a significant positive effect on academic performance in model 2. All tolerances were acceptable in the regression models. Model 1 and model 2 explained 10.4% and 12.4% of variance in academic performance respectively.

Consequently, cultural distance and gender showed no significant effect on academic performance. The male and female students showed no significant difference in academic performance, although they presented differential cultural adaption ability after crossing the border (Paglin & Rufolo, 1990; van de Werfhorst, 2002). The result did not support H1 that cultural distance negatively influences academic performance. However, this result supported H2 that female gender negatively moderates the contribution of cultural distance to academic performance, as supported by role theory (Allen et al., 2002; Dindia & Canary, 2006). Therefore, it refuted H1 but supported H2 regarding the moderating effect. Furthermore, the female student faced more difficulty to adapt to the new culture and thus to perform well academically than did the male one compared to male students.

## Discussion and implication

In relation to perceived cultural distance to academic performance in cross-border students (Hagedorn & Tierney, 2002; Hoffman & Lowitzki, 2005), previous literature (Martin et al., 2012; Martin et al., 2013) indicates that stating that cultural distance does not affect students’ academic performance after crossing the border is difficult to say. Thus, we considered that there must be some moderators between cultural distance and academic performance, for example, gender (Francis & Penny, 2014). Women are sensitive to environmental change (Dupuis et al., 2008), which hinders their performance after crossing the border. Sociocultural factors in gender roles and socialization patterns (Francis & Penny, 2014; Levitt, 1995; Mol, 1985) may impede women’s social interaction and cooperation. The effect of gender in this study is one of the examples that males present a positive effect in the relationship between cultural distance and academic performance, whereas female students show the opposite.

Furthermore, like Deaner (2006), we found gender difference in performance, which also expands Deaner’s (2006) work in student groups. This phenomenon presented in many studies and was corroborated by Frick (2011), who showed similar patterns to test the sex difference in relative performance. Therefore, this study examined the effect of gender in the relationship between cultural distance and academic performance. As hypothesized, an interaction between gender and cultural distance exists. Gender influences the effect of cultural distance on academic performance in many ways. Female cross-borderers showed lower cultural adaptation compared with their counterparts. Several reasons result in this phenomenon. First, women have fewer resources for decreasing cultural distance and supporting their lives after crossing the border (Xie & Shauman, 2003). Second, high cultural distance may involve the consideration of safety, basic survival needs, and welfare, which are concerns of women (Dupuis et al., 2008); thus, they cannot fully focus on their study or career. Women are sensitive to environmental changes (Dupuis et al., 2008), which hinders their performance after crossing the border. Third, women might be less interactive and cooperative (Francis & Penny, 2014; Levitt, 1995; Mol, 1985).

The findings warrant the application of role theory to academic performance in a cross-border study. Role theory has been applicable to guiding research on interaction (Allen et al., 2002; Dindia & Canary, 2006; Eagly, 1987), resulting in cultural distance after migration. The theory can help predict attitudes and behaviors in society with reference to the sociocultural context (Chatillon et al., 2018). Women communicating through indirect means rather than direct means (Fischer, 2004) increase the distance between the host culture and their own culture. Males have more economic capital and quantitative skills for thinking of getting higher grades for their long-term benefits, such as winning scholarships.

The study’s findings may be particularly important to policymaking for cross-border students, especially in “Talent Plans.” Compared to international migration (Yang & Qin, 2016; Yue et al., 2016, p.79), the sample of cross-border students in universities contributes to filling the research gap of internal migration or cross-border. Governments can reduce gender differences in perceived cultural distance among cross-border students, for instance, by organizing cultural exchange activities for female students particularly. In addition, the study suggests enhancing the confidence of administrators or policymakers in providing considerably needed support for female cross-border students. For example, programs for newcomers for cultural exchange can pay attention to female students. The results also suggest universities and teachers to provide resources and encouragement, particularly to female students to improve their cultural adaptation and academic performance successively. In addition, based on role theory, women should overcome difficulties in adapting to the host society and perform well (Shimanoff, 2009). These difficulties might result from the female characteristic of being sensitive to cultural distance (Charles & Bradley, 2002; Charles & Bradley, 2009).

## Limitation and future research direction

First, despite their common use in educational research (Pace, 1985), self-reported data (GPA and prizes) has biases, which require clear phrasing of questions and students’ careful mind in responding to the questions (Pace, 1985). Anyhow, self-report measures are at risk of subjective bias (Nederhof, 1985). This must be a reflection point of school-reported measures. Therefore, future research should incorporate more objective measures.

Second, student achievement in the context of ability (e.g., team spirit and leadership) is considerably broader than test scores, which should include self-knowledge, exhibits, portfolios, etc. (Lambert, 2003, p. 7). Academic performance that only includes GPA and prizes cannot sufficiently explain academic performance. Thus, other measurements of teamwork, leadership, etc., will be helpful in future studies.

Third, some unmeasured variables might confound the gender differential and other findings. Future research needs to identify such variables and examine their confounding effects.

Finally, this study only included participants in China. Samples from other places are necessary to clarify the effects cross-culturally. Comparison of different cultures is necessary to ascertain the robustness of the present findings.

## Conclusion

The present study demonstrated gender difference in the effect of cultural distance on academic performance among cross-border students in Mainland China. While Western research mainly paid attention to international migration (Yang & Qin, 2016; Yue et al., 2016, p.79), the data of this study, cross-border students in universities, contributes to filling the research gap of migration research.

Likewise, the demonstration of the nature in the relations of cultural distance and academic performance further stresses the need to foster the values utilization of the host culture and the acceptance of a bicultural position in host communities. Women are sensitive to cultural change, and lacking resources in the society makes them face more difficulties in adapting to the changing culture, which influences their performance given that they cannot focus on their studies. Furthermore, governments and universities should pay more attention to the lower cultural adaptation groups, that is, female cross-borderers.

Self-reported data, the measurement of academic performance, other unmeasured variables which might confound the gender differential, and the limitation of samples (only Chinese samples included) hindered the accuracy of the results, which should be improved in the future studies. For instance, incorporating more objective measures, adding teamwork and leadership to the measurement of academic performance, identifying gender differential variables, and examining their confounding effects and comparison of different cultures with cross-border samples in diverse countries should be considered in the future studies.

## Data Availability

No
